# The Burden of Hepatitis B, Hepatitis C, and Human Immunodeficiency Viruses in Ovarian Cancer Patients in Nairobi, Kenya

**DOI:** 10.3390/idr14030047

**Published:** 2022-06-07

**Authors:** Francis Mugeni Wanyama, Rudolf Tauber, Alfred Mokomba, Catherine Nyongesa, Véronique Blanchard

**Affiliations:** 1 Institute of Laboratory Medicine, Clinical Chemistry and Pathobiochemistry, Charité—Universitätsmedizin Berlin, Corporate Member of Freie Universität Berlin, Humboldt-Universität zu Berlin and Berlin Institute of Health, Augustenburger Platz 1, D-13353 Berlin, Germany; rudolf.tauber@charite.de; 2 Clinical Chemistry Unit, Department of Human Pathology, University of Nairobi, P.O. Box 19676, Nairobi 00202, Kenya; 3 Labor Berlin Charité Vivantes GmbH, Sylterstrasse 2, D-13353 Berlin, Germany; 4 Department of Obstetrics and Gynecology, Kenyatta National Hospital, Ngong Road, P.O. Box 20723, Nairobi 00202, Kenya; mokombadoc@gmail.com; 5 Cancer Treatment Centre, Kenyatta National Hospital, Ngong Road, P.O. Box 20723, Nairobi 00202, Kenya; cnyongesa@knh.or.ke; 6 Texas Cancer Center, Keri Road Off Mbagathi Way, P.O. Box 13, Nairobi 00202, Kenya; 7 Department of Human Medicine, Medical School Berlin, Rüdesheimer Straße 50, D-14197 Berlin, Germany

**Keywords:** ovarian cancer, hepatitis B virus, human immunodeficiency virus, hepatitis C virus, seroprevalence, chemotherapy, Kenya, sub-Saharan Africa

## Abstract

Ovarian cancer (OC) is a gynecological malignancy characterized by high morbidity and mortalities due to late-stage diagnosis because accurate early diagnostic biomarkers are lacking. Testing of Hepatitis B virus (HBV), Hepatitis C virus (HCV), and Human immunodeficiency virus (HIV) infections in OC patients is pertinent in light of the emerging evidence of their contribution to poor prognosis. We, for the first time, investigated the prevalence of HBV, HCV, and HIV infections in a Kenyan cohort of OC to inform optimal management. We recruited a cohort of women above 18 years of age, comprising 86 OC patients and 50 healthy controls. Participants’ blood samples were serologically screened for HBV, HCV, and HIV. We found seroprevalence rates of 29.1%, 26.7%, and 1.2% for HBV, HIV, and HCV, respectively, in OC patients. The healthy control group had HBV and HIV seroprevalence rates of 3.9% for each with no positive HCV case. HBV/HIV coinfection was noted only in the OC group with a positivity rate of 17.4%. In summary, we found higher HBV and HIV seroprevalence in Kenyan OC patients compared to the healthy control group, whereas HCV prevalence was reflective of the general population. Hence, we recommend screening for HBV and HIV among OC patients destined for anticancer treatment.

## 1. Introduction

Ovarian cancer (OC) is a lethal gynecological malignancy that has aggressive progression resulting in high fatality rates worldwide. It is the eighth major cause of cancer death in women, with the highest incidence rates of >7.5 per 100,000 reported in the more developed regions and the lowest of <5 per 100,000 in sub-Saharan Africa [1]. Previous findings from Kenya, a sub-Saharan country, showed that OC is the third most common cause of cancer death among gynecological cancers [2]. Late diagnosis due to lack of effective early diagnostic biomarkers, unspecific symptoms in the early stages of the disease, and the propensity of the malignant cells to develop chemo-resistance, are the major contributors to high mortalities in OC [3]. Although research on early biomarkers for OC has intensified in the past decades, the time of diagnosis is yet to improve OC [4,5,6]. Infectious agents such as HBV, HIV, and possibly HCV have also been associated with early deaths of cancer patients including OC if not well accounted for during patient management [7,8]. Only a few studies have attempted to determine the burden of HBV, HCV, and HIV infections in OC patients globally, and to the best of our knowledge, none in the sub-Sahara African region and Kenya to be specific.

Infectious agents that principally contribute to the global cancer burden are *Helicobacter pylori* (5.5%), human papillomaviruses (5.2%), HBV, and HCV (4.9%), Epstein-Barr virus (1%), HIV and human herpesvirus (0.9%) [9,10]. GLOBOCAN 2018 and the African-based population registries reported human papillomaviruses as the major contributor of infection-based cancers in Africa at 12.1% and 15.4% in sub-Sahara Africa, with cervical cancer being the most common. Others are Kaposi sarcoma-associated virus (3.1%), HBV, and HCV (2.9%), *Helicobacter pylori* (2.7%), Epstein-Barr virus (2%), and *Schistosoma haematobium* causing bladder cancer (1%) [11].

Hepatitis B virus is a member of Hepadnaviruses family, known to be endemic in parts of Africa and Asia. The sub-Saharan Africa region is classified as highly endemic with an overall HBV prevalence of ≥8% [12,13], whereas Asia also has a high prevalence, ranging from 8–12% [14]. Previous findings from Kenya showed varying prevalence rates of HBV depending on the geography of the study area. For instance, an HBV prevalence of 8.8% was reported in a northern rural population, whereas another study reported a prevalence of 11.2% in the eastern region of Kenya [15,16]. Reports from other sub-Saharan African countries neighboring Kenya show a similar pattern. For instance, Bwogi et al. reported that Ugandan HBV prevalence varied geographically but with a consolidated national prevalence of 10% [17]. In Burundi and Ethiopia, similar HBV prevalence rates of 11% were reported [18,19]. A Hong Kong study reported a 10.1% prevalence of HBV in primary OC patients and also associated its chronicity with reduced survival of the patients [7]. Individuals infected with HBV are at increased risk of developing liver cirrhosis, deterioration of hepatic function (liver decompensation), and eventually hepatocellular carcinoma (HCC) [20]. HBV is also an important cause of poor prognosis among OC patients with current or resolved HBV infection [7]. Ovarian cancer patients with initially resolved HBV are at an increased risk of HBV reactivation when subjected to anti-cancer treatment without additional antivirals [21,22].

Although the pathophysiological mechanisms through which HBV infection influences OC patients’ prognosis are yet to be overtly described, a few possible contributing factors have been suggested. Factors thought to influence the poor survival of HBV-infected OC patients include modulation of the immune system, which compromises the immune surveillance mechanism responsible for host anti-cancer cell response and virus-specific adaptive immunity [23,24]. Hepatitis delta virus (HDV), an obligate satellite of HBV that infects humans concomitantly with or after HBV infection [25], is also an important contributor to poor patient outcomes. The superinfection of HDV in chronically HBV-infected individuals often results in a severe form of viral hepatitis with faster progression to cirrhosis and HCC compared to the HBV mono-infection [26]. Thus, OC patients co-infected with HBV and HDV are likely to experience a further decline in longevity. However, reports indicate that HDV affects only 5% of the chronic HBV cases globally, and the prophylactic vaccine for HBV simultaneously protects against HDV since the latter relies on HBV for packaging and transmission [26,27].

About 50–85% of patients with HCV infection proceed to develop chronic hepatitis in 20–30 years’ time [28,29]. HCV prevalence in Kenya’s general population is relatively low compared to other parts of Africa. Recent studies in Kenya reported rates of 0.2–1.8% among blood donors, and 22% for drug users [30,31]. Globally, Egypt has the highest national prevalence of HCV at 14.7% in people aged 15–59 years [32]. HCV infection often leads to liver cirrhosis, hepatocellular cancer, liver failure, and death [33]. Therefore, just as in the case of HBV, HCV infection in OC patients aggravates the poor prognostic situation of OC disease.

HIV infection has also been associated with poor outcomes in gynecological cancer patients [34,35]. Over two-thirds of the 34 million people living with HIV globally reside in sub-Saharan Africa [36]. Kenya, which is sub-Saharan country, has the latest published HIV prevalence rate of 4.5% in people aged 15–49 years [37]. Kaposi’s sarcoma, cervical cancer, and Hodgkin lymphoma are the major cancers defining Acquired Immune Deficiency Syndrome (AIDS) but are currently on the decline due to the widespread use of antiretroviral therapy [35]. A consequence of the decline in cases of AIDS is that non-AIDS-defining gynecologic malignancies are rising because of the patients’ improved longevity and aging [35,38,39]. Reports from previous studies have shown an association of HIV-seropositive status with poor prognosis of gynecologic cancer patients when proper management strategies are not implemented [8]. An American study, for instance, reported worse survival of HIV-infected women with advanced gynecologic cancers who did not receive adherent care by the National Comprehensive Cancer Network [8]. The use of antiretroviral drugs, proteasome and protease inhibitors, are beneficial to HIV-infected OC patients as they enhance the susceptibility of drug-resistant OC cells to anti-cancer agents [3,40].

About 10% of the people infected with HIV worldwide have chronic HBV infection [41], which results in higher morbidity and mortalities compared to mono-infections of either type [42]. Seroprevalence rates of 4.26% and 0.46% for HBV/HIV and HIV/HCV, respectively, were reported in a Kenyan population living in an urban informal settlement [43]. The major challenge of HBV/HIV coinfection is that HIV causes immunosuppression leading to reduced HBV clearance [44], whereas HBV, in a reciprocal manner, blunts the effectiveness of the antiretroviral drugs [45,46], all adding up to compromised immune surveillance against cancer cells. In the quest for effective management of OC patients co-infected with HBV/HIV, research has implied the use of nucleoside analogs as the best alternative because they are active against both HIV and HBV. For instance, the use of tenofovir disoproxil fumarate and emtricitabine as first-line antiretroviral therapy for HIV also treats HBV by default [42].

Currently, screening for HBV, HCV, and HIV is not routinely done in OC patients scheduled for anti-cancer treatment in many of the health facilities in Kenya, unlike in some countries such as Hong Kong and the United States where it is a requirement [7,8]. With only a 50% survival of OC patients in two years in Kenya, inadequate management of OC patients infected with HBV, HIV, or HCV is likely to be an important contributor to their greatly diminished life span hence very pertinent to define the burden. To the best of our knowledge, no study within Kenya or the greater sub-Saharan region has determined the seroprevalence rates of HBV, HCV, HIV, and their coinfections among OC patients. We therefore, for the first time, determined the seroprevalence rates of HBV, HCV, HIV, and their coinfections in a cohort of Kenyan OC patients and compared them with a healthy control to estimate the burden. The rationale was to define the problem and justify the need to integrate routine screening of HBV, HCV, and HIV in the diagnosed OC patients scheduled for chemotherapy, for improved patient prognosis.

## 2. Materials and Methods

### 2.1. Recruitment of Study Participants

In this descriptive study, we recruited a cohort of 86 adult OC patients and 50 healthy control women from April 2018 to April 2020. Of the 86 OC patients, 19 patients were primary, whereas 67 had received a varying number of chemotherapy cycles. All the subjects consented to participate in the study. The subjects were recruited in a multi-center setting within Nairobi, Kenya, as per the ethical vote obtained from Kenyatta National Hospital / University of Nairobi ethics review committee (KNH/UON-ERC) reference no P701/12/2017. The recruiting centers were Kenyatta National Hospital (KNH), St. Mary’s Hospital Langata, and the Texas Cancer Centre. The samples collected were analysed at Charité-Universitätsmedizin, Berlin, Germany.

### 2.2. Blood-Serum Sample Collection

Venule blood from the participants’ was collected into 5 mL vacutainers with a serum clot activator (Becton, Dickinson GmbH, Heidelberg, Germany). Blood samples in the vacutainers were allowed to stand at room temperature for 30 min to 2 h to allow the clot to retract, followed by centrifugation at 1200× *g* for 15 min. Then serum was aliquoted into the Eppendorf tubes, then stored at −80 °C until their batch shipment to Berlin, Germany, upon getting the necessary approvals from the responsible agencies in Kenya and Germany. The Kenyan approval documentation included: KNH/UoN-ERC Ref no., KNH-ERC/shipment/40, Ministry of Health, letter of no objection Ref. No. MOH/F/HRD/01/VOL.11, and the Kenya Pharmacy and Poisons board export permit Ref. no. CD2021000PPB321J0002550623. The ethical commission of the Charité-Universitätsmedizin, Berlin, Germany, granted the use of the samples for analysis (approval number EA4/071/19).

### 2.3. Laboratory Analysis of HBV, HCV, and HIV

Serological measurements of HBV (Hepatitis B surface antigen, HBsAg), HCV (anti-HCV), and HIV (simultaneous qualitative detection and differentiation of HIV-1 p24 antigen and antibodies to HIV-1 (groups M and O) and HIV-2) were done using a Cobas e 801 immunoassay system (Roche Diagnostics GmbH, Penzberg, Germany). Cobas e 801 immunoassay system is a high throughput immunochemistry module designed to carry out fully automated electrochemiluminescence immunoassays. The reagents used in this analysis were Elecsys HBsAg II, Elecsys anti-HCV II, and Elecsys HIV Duo (HIV-Ag and anti-HIV) reagent (Roche Diagnostics GmbH, Penzberg, Germany). 

### 2.4. Data Management

The OC patients and the control subjects were stratified according to their HBV, HCV, and HIV serological status (seropositive and seronegative). The relationship between the subjects’ serological status with their baseline demographic and clinical factors was then analyzed using various statistical tests as appropriate. Demographic and clinical factors included age, tumor stage categorized as early (FIGO stage II & I) or late (FIGO stage III & IV) stage, the surgical status of the tumor, and the status of chemotherapy intake. Continuous variables were presented as a range whereas qualitative data were captured as numbers and percentages. Non-continuous variables were compared by Chi-square test, Fisher’s exact test, two proportions Z test, and Mann–Whitney U test as appropriate. The odds ratios (OR) were also determined for risk estimation. The level of significance was set at *p* < 0.05. Data were analyzed using SPSS version 25 (SPSS, Chicago, IL, USA).

## 3. Results

We recruited 86 OC patients and 50 healthy control women after meeting inclusion criteria. Later, we determined their seropositivity status (positive tests) for HBV (HBsAg), HCV (anti-HCV), HIV (simultaneous qualitative detection and differentiation of HIV-1 p24 antigen and antibodies to HIV-1 (groups M and O) and HIV-2), and the possible coinfection status (Supplementary Tables S1 and S2). We then compared the HBV, HCV, HIV, and HBV/HIV coinfection seroprevalence rates of OC patients to the control subjects. Further, we analyzed the distribution of seropositivity states according to the subjects’ age, age classification, and clinical factors at enrollment. Classification of the age of the subject was based on the Kenyan menopausal age reported earlier as 48.28 years by Noreh et al. [47]. Therefore, the age of subjects was classified into two, premenopausal (18–48 years) and postmenopausal (≥49 years). On the other hand, the clinical factors included tumor stage (late or early), cancer management approaches of surgical debulking (surgical debulking done or not done), and chemotherapy (on chemotherapy or not).

### 3.1. Description of Participants’ Baseline Demographic and Clinical Factors

The participants were initially described by their age and clinical variables at enrollment (Table 1). The OC patients’ age ranged from 19–81 years with a median of 50 years and an interquartile range of 26, whereas for the control group, their age ranged from 18–74 years with a median of 34 years and an interquartile range of 18. Patients with early tumor stages were 17.4%, whereas 86.6% were in the late (advanced) stage. Additionally, at the time of enrollment, 44.2% of the patients had undergone operative debulking whereas 55.8% had not. Moreover, 77.9% of the patients were on chemotherapy whereas 22.1% had not started chemotherapy.

### 3.2. Comparison of HBV, HCV, HIV, and HBV/HIV Serological Status of the Ovarian Cancer Patients against the Control Subjects

**Figure 1:**
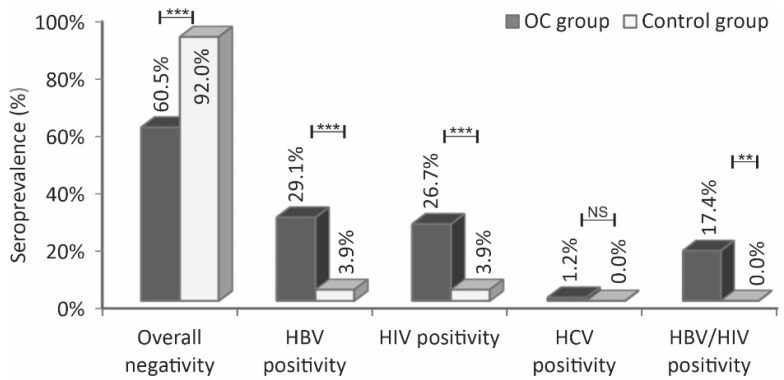
Comparison of HBV, HCV, HIV, and HBV/HIV seroprevalence rates in ovarian cancer patients against the control group and the overall seronegativity (HBV + HCV + HIV) of the OC patients against the control. Statistical significance was indicated as ** *p* ≤ 0.01 and *** *p* ≤ 0.001, analyzed by two proportion Z test.

The differences in the distribution of HBV, HIV, and HBV/HIV seropositivity status between the OC patients and the controls were significant (HBV and HIV, *p* < 0.001; HBV/HIV, *p* = 0.002), whereas HCV seropositivity was not significant (*p* = 0.441) (Table 2). The seropositivity rates for the OC patients were 29.1% (HBV), 26.7% (HIV), 1.2% (HCV), and 17.4% (coinfection of HBV/HIV). Conversely, the HBV and HIV seropositivity rates for the control group were 3.9% each. Additionally, the overall (HBV, HCV, and HIV) seronegativity for OC patients and the control group were 60.5% and 92%, respectively (Figure 1). The control group had no positive case for HCV or HBV/HIV coinfection.

### 3.3. Association of HBV, HCV, HIV, and HBV/HIV Serological Status of the Participants with Age

Analysis of the OC patients’ age distribution in the seropositive and seronegative states of HBV, HCV, HIV, and HBV/HIV coinfection showed no significant difference (HBV, *p* = 0.266; HIV, *p* = 0.575, HCV, *p* = 0.442 and HBV/HIV, *p* = 0.153) (Table 2). However, the age difference between the HBV seropositive control subjects and the seronegative control subjects was significant (*p* = 0.026), but not so for the difference in age among the HIV-seropositive and seronegative control subjects (*p* = 0.320). The median age for seropositive against seronegative OC patients was 52 vs. 49 for HBV, 49 vs. 51 for HIV, and 55 vs. 49 for HBV/HIV coinfection. For the control, the medians for the seropositive against the seronegative were 54.5 vs. 34 and 44.5 vs. 34 for HBV and HIV, respectively.

### 3.4. Association of HBV, HCV, HIV, and HBV/HIV Seropositivity Status with the Subjects’ Age Groups (Menopausal Status)

Evaluation of the relationship between OC patients’ age groups and the seropositivity status of the three markers yielded diverse results (Table 3). The OC patients of age 49 years and above showed higher odds for seropositive HBV, HCV, and the coinfection of both, compared to patients in the age bracket of 18–48 years [HBV, OR 1.611 95% CI (0.618–4.203); HCV, OR 1.027 95% CI (0.975–1.082) and HBV/HIV, OR 2.527 95% CI (0.735–8.693)]. Conversely, OC patients aged 49 years and above were less likely to be HIV-seropositive compared to patients in the age bracket of 18–48 years, OR 0.754 95% CI (0.285–1.994). On the other hand, analysis of the control subjects showed higher odds of HBV, and HIV seropositivity in women aged 49 years and above compared to those in the age bracket of 18–48 years, HBV; OR 5.000 95% CI (0.282–88.527) and HIV; OR 1.051 95% CI (0.981–1.127).

### 3.5. Association of Ovarian Cancer Patients’ HBV Serological Status with Clinical Factors

Of the 29.1% (25) OC patients who tested positive for HBV, the odds for seropositivity were higher among patients in the late tumor stage, (OR 1.155, 95% CI, 0.330–4.042), and those that had done surgical debulking, (OR 1.243 95% CI, 0.488–3.163) compared to their respective counterparts (early tumor stage and not done surgical debulking) (Table 4). However, OC patients who were not on chemotherapy were less likely to test positive for HBV compared to those on chemotherapy treatment (OR 0.858 95% CI, 0.284–2.586). The difference in the distribution of the HBV serological status in the patients’ clinical factors did not attain statistical significance (tumor stage, *p* = 1.000; surgical debulking, *p* = 0.648 and chemotherapy, *p* = 0.785). 

### 3.6. Association of Ovarian Cancer Patients’ HIV Serological Status with Clinical Factors

Of the 26.7% (23) HIV seropositive cases screened, a majority were reflected in the categories of late-stage tumor OC (OR 2.730 95% CI 0.566–13.168) and those that had undertaken surgical debulking (OR 1.222 95% CI 0.469–3.187) in comparison to their respective opposite counterparts (Table 5). The odds for HIV seropositivity were lower among OC patients that were not on chemotherapy compared to those who were on chemotherapy treatment (OR 0.858 95% CI 0.242–2.242). A comparison of the distribution of HIV seropositive and seronegative status of the OC patients’ in their clinical variables revealed no significant difference (tumor stage, *p* = 0.335; surgical debulking, *p* = 0.681 and chemotherapy, *p* = 0.590).

### 3.7. Association of Ovarian Cancer Patients’ HBV/HIV Coinfection Status with the Clinical Factors

A higher frequency of HBV/HIV coinfection positivity was exhibited in the OC patients’ categories of the advanced-stage tumor, (OR 1.457 95% CI 0.292–7.257), patients that had done surgical debulking, (OR 1.562 95% CI 0.510–4.779) and patients who were not on chemotherapy treatment (OR 1.279 95% CI 0.491–3.329), compared to their opposite counterparts (Table 6). Comparison of the distribution of OC patients’ HBV/HIV co-infection status of seropositivity and seronegativity with respect to the patients’ clinical factors revealed no significant difference (tumor stage, *p* = 1.000; surgical debulking, *p* = 0.432 and chemotherapy, *p* = 0.733).

## 4. Discussion

Ovarian cancer remains one of the most common causes of death among gynecological malignancies afflicting women worldwide [2]. Infectious agents such as HBV, HIV, and HCV weigh on the already weak immune system of the OC patients’, causing further contraction to survival if management is not adequate. For the first time, we document the seroprevalence rates of HBV, HIV, and HCV in a Kenyan cohort of ovarian cancer patients and the healthy control women. From our findings, OC patients’ had HBV, HIV, and HCV seroprevalence rates of 29.1%, 27%, and 1.2%, respectively. In addition, a coinfection of HBV and HIV was reported with a prevalence of 17.4%. Comparatively, the HBV and HIV seroprevalence rates of OC patients’ in the current study were significantly higher than that of the healthy control group which posted a seroprevalence of 3.9% for both HBV and HIV, with no positive case of HCV or coinfection reported. Findings on the seroprevalence rate of HBV among the healthy control group were in keeping with the rates previously reported in Kenyan and Ethiopian obstetrics populations which had 3.8% and 3.7%, respectively [48,49]. We found a lower HIV prevalence (3.9%) among the healthy control group compared to the earlier reported prevalence of 4.5% in 2019 by UNAIDS [37]. Our control data was not sufficient to conclude that there was a general reduction in the number of people contracting HIV in Kenya, much as the Kenyan government and other non-governmental agencies have escalated the control measures to address the disease. Important to note also, was that the seroprevalence rate of HBV/HIV coinfection among OC patients was higher compared to the 4.26% seroprevalence rate reported by an earlier study done on a Kenyan population living in an urban informal settlement [43]. Additionally, another study involving 159 HIV-positive female sex workers in Mombasa, Kenya, who were receiving long-term antiretroviral therapy had also reported a lower HBV prevalence rate of 6.9% compared to the findings of the present study [50].

Ovarian cancer patients had higher seropositivity rates of HBV and HIV compared to the previous findings on Kenyan non-ovarian cancer populations [12,15,16,51]. Moreover, a higher rate of HBV/HIV coinfection among OC patients was also noted in contrast to the rate of 4.2% previously reported from a population living in an informal urban settlement in Kenya [43]. The differences in findings between the current and previous studies could be ascribed to selection bias based on the nature of the study population and the setting. Selection bias, for instance, was earlier shown by a study done at a liver clinic of a National Hospital in Nairobi, Kenya, that reported HBsAg positivity of 77% in patients that had chronic aggressive hepatitis or cirrhosis [52]. Unlike the previous studies, the present study evaluated OC patients who are amenable to viral infections due to the state of immune depression resulting from malignant activities and anti-cancer chemotherapy. Furthermore, the study subjects were enrolled in a hospital setting and hence had an increased susceptibility to nosocomial viral infections due to frequent patient hospitalizations during surgery and chemotherapy. On the other hand, the seropositivity rate of HCV was concordant with previous Kenyan studies that reported rates of 0.2–1.8% [30,43]. Although earlier findings had found no effect on the overall survival of OC patients by the compensated liver cirrhosis due to HCV [29], more studies in endemic areas are required to corroborate this finding.

In the current study, 77.9% of the OC patients were on chemotherapy and, with a likelihood that a part of them had a current and others clinically resolved HBV infection. Therefore, the high HBV seropositivity rate reported could not only be because of the active infection but also a possible consequence of relapse due to the lack of routine testing within the study setting for the HBV infection in the OC patients to guide optimal patient management. Findings from earlier studies had indicated that patients with initially resolved HBV are at higher risk of HBV reactivation when they are put on anti-cancer chemotherapy agents without additional antivirals [21,22]. Therefore, for optimal management of cancer patients, greater benefit is achieved when testing of HBV in OC patients is done prior to anti-cancer treatment. The American Society of Clinical Oncology (ASCO) recommended testing of HBV for all patients expecting anti-cancer treatment and further proposed a risk-adaptive clinical algorithm to guide the treatment of cancer by minimizing the risks of HBV relapse among those with chronic HBV [53].

There also appeared to be an association between HBV and HIV seropositivity states with the OC patients’ age and their clinical factors. The older patients had higher odds for HBV positivity compared to the younger group. A higher state of HBV positivity among elderly patients is likely to be due to the fact that they were exposed to the risk of HBV infection for a longer period of time. Conversely, the young OC patients appeared more likely to test positive for HIV compared to the older patients. This is because young women are more sexually active compared to the elderly and therefore highly at risk of infection since HIV is largely transmitted through sexual activities. Although the association of HBV seropositivity with the patients’ clinical factors was not significant, the propensity of HBV seropositivity was higher in the OC patients who were in the advanced tumor stage and those that had undergone surgical debulking. The higher seropositivity rate of HBV among patients who had done surgical debulking might partly be attributed to nosocomial transmissions since the procedure of surgical debulking is usually accompanied by hospitalization. On other hand, higher HBV positivity among the advanced stage tumor patients could partly be ascribed to the possibility of relapse due to depressed immunity associated with cancer metastasis and chemotherapy. Our findings on HBV seropositivity were in agreement with the previous report on a Hong Kong OC cohort that reported higher odds of HBV seropositivity in advanced OC, a state that correlated with poor patient survival [7]. Modulation by HBV infection of the immunological mechanisms involved in tumor rejection may be a factor responsible for the observed association [24].

Previously, HIV infection was reported to negatively impact the prognosis of patients with gynecological cancers [54]. Our findings showed higher odds of HIV seropositive status among patients with advanced-stage OC and those that had done surgical debulking. From reports of previous studies, HIV-infected OC patients could optimally be managed by a two-pronged strategy that both suppresses the viral load and also curtails the cancer aggressiveness, using HIV antiretroviral (ART) drugs and anti-cancer agents [3,40,55]. Interestingly, Nelfinavir, a protease inhibitor ART, acts on both carboplatin-sensitive and resistant ovarian cancer cells by inducing apoptosis and inhibiting proliferation [40,56,57]. This means that OC patients with HIV infection can benefit from the use of the Nelfinavir ART not only to reduce the viral load but also to eradicate the OC resistant cells, thereby curtailing progression. Similarly, a combination of Ritonavir ART and paclitaxel, a chemotherapy agent for OC, were reported to have an anti-proliferating effect against OC cells [3].

Although our findings on the association of the HBV/HIV coinfection status of OC patients with clinical factors did not show a significant difference, higher frequencies of seropositivity were found in patients who were in the late stage of the tumor disease, done surgical debulking, and not on chemotherapy. HIV has been reported to affect the natural history of HBV causing spontaneous reverse seroconversion to an active HBV state [42]. A South African study on HIV/HBV coinfection management recommended the use of Tenofovir as the alternative to Lamivudine, which had not shown an impact on reducing mortalities [58]. Tenofovir disoproxil fumarate and emtricitabine also were shown to not only treat HIV but also treat HBV by default [59]. The limitation of the present study was that we could not quantify the impact on survival of the OC patients whose infectivity status was not known during treatment vis-a-vis those with known status. Therefore, we recommend a retrospective or controlled progressive study to ascertain the impact of HBV and HIV on the survival of OC patients in Kenya and the larger sub-Saharan African region, which is an endemic area of HIV and HBV.

## 5. Conclusions

From our findings, we conclude that Kenya has a higher burden of HBV and HIV seroprevalence in ovarian cancer patients, but the seroprevalence rate of HCV mirrors that of the general population. Therefore, given the findings of the present study, we recommend routine testing of HBV and HIV in OC patients prior to administration of chemotherapy to allow selection of the best treatment strategies that will suppress the viral load as well as curtail tumor growth and consequently enhance patients’ longevity. On the other hand, testing of HCV should be done only on a needs basis, given the comparatively low prevalence.

## Figures and Tables

**Table 1:** Description of participants’ baseline demographic and clinical factors.

Characteristics	OC Group n = 86	Control Group n = 50
Age (years)		
Range	19–81	18–74
Mean ± SD	50.6 ± 15.6	37.9 ± 11.5
Median	50	34.5
Interquartile range	39–65	29–47
Tumor stage % (n)		
FIGO I & II (early stage)	17.4 (15)	
FIGO III & IV (late stage)	82.6 (71)	
Surgical debulking % (n)		
Yes	44.2 (38)	
No	55.8 (48)	
Chemotherapy % (n)		
On chemotherapy	77.9 (67)	
No chemotherapy	22.1 (19)	

Age is in years, whereas the clinical factors are presented in percentages and total count (percentage).

**Table 2:** Association of HBV, HCV, HIV, and HBV/HIV serological status of the participants with age.

Infection	OC Group, n = 86			Control Group, n = 50		
	Seropositivity	Seronegativity	*p*-Value	Seropositivity	Seronegativity	*p*-Value
HBV						
Age range (years)	19–74	20–81	0.266 ^b^	50–59	18–74	0.026 ^b^
Mean ± SD	52.8 ± 16.1	49.7 ± 15.4		54.5 ± 6.4	37.2 ± 11.2	
Median	52	49		54.5	34	
Interquartile range	41–68	38–61		-	28–46	
HIV						
Age range (years)	28–73	19–81	0.575 ^b^	42–47	18–74	0.320 ^b^
Mean ± SD	51.9 ± 12.9	50.1 ± 16.5		44.5 ± 3.5	37.6 ± 11.6	
Median	49	51		44.5	34	
Interquartile range	45–60	38–65		-	28–46	
HCV						
Age range (years)	38	19–81	0.442 ^b^	-	18–74	
Mean ± SD	38	50.7 ± 15.6		-	37.9 ± 11.5	
Median	-	50.7		-	34.5	
Interquartile range	-	39–65		-	29–47	
HBV/HIV						
Age range (years)	19–74	20–81	0.153 ^b^	-	18–74	
Mean ± SD	55 ± 13.7	46 ± 15.8		-	37.9 ± 11.5	
Median	55	49		-	34.5	
Interquartile range	48–69	38–63		-	29–47	

Analysis of the participants age distribution in HBV, HCV, HIV, and HBV/HIV sero-status presented in mean ± standard deviation, range and *p*-value (<0.05). ^(b)^ Mann–Whitney U test.

**Table 3:** Association of HBV, HCV, HIV, and HBV/HIV seropositivity status with the menopausal status.

Sero-Status % (n)	OC Group, n = 86				Control Group, n = 50			
	18–48 Years	>49 Years	OR (95% CI)	*p*-Value	18–48 Years	>49 Years	OR (95% CI)	*p*-Value
HBV								
Seropositivity	36 (9)	64 (16)	1.611 (0.618–4.203)	0.327 ^c^	50 (1)	50 (1)	5.000 (0.282–88.527)	0.230 ^d^
Seronegativity	47.5 (29)	52.5 (32)			83.3 (40)	16.7 (8)		
HIV								
Seropositivity	39.1(9)	60.9 (14)	0.754 (0.285–1.994)	0.568 ^c^	100 (2)	0 (0)	1.051 (0.981–1.127)	0.496 ^d^
Seronegativity	46 (29)	54 (34)			81.3 (39)	18.8 (9)		
HCV								
Seropositivity	100 (1)	0 (0)	1.027 (0.975–1.082)	0.258 ^d^	-	-	-	-
Seronegativity	43.5 (37)	56.5 (48)			82 (41)	18 (9)	-	
HBV/HIV								
Seropositivity	26.7 (4)	73.3 (11)	2.527 (0.735–8.693)	0.134 ^d^	-	-	-	-
Seronegativity	47.9 (34)	52.1 (37)			82 (41)	18 (9)	-	

The data shows analysis of the subjects’ seropositivity status of HBV, HCV, HIV, and HBV/HIV in their respective age groups as presented in the form of percentages and counts (in brackets), % (n), odds ratio, 95% confidence interval (OR, 95% CI) and *p*-value (<0.05). ^(c)^ Chi-square and ^(d)^ Fisher’s exact test.

**Table 4:** Association of ovarian cancer patients’ HBV serological status with their clinical factors.

Clinical Factors	HBV Seropositive 29.1% (25)	HBV Seronegative 70.9% (61)	OR (95% CI)	*p*-Value
Tumor stage % (n)			1.155 (0.330–4.042)	1.000 ^c^
Late stage	24.4 (21)	58.1 (50)		
Early stage	4.7 (4)	12.8 (11)		
Surgical debulking % (n)			1.243 (0.488–3.163)	0.648 ^c^
Done	14 (12)	30.2 (26)		
Not done	15.1 (13)	40.7 (35)		
Chemotherapy % (n)			0.858 (0.284–2.586)	0.785 ^c^
No chemo	7 (6)	15.1 (13)		
On chemo	22.1 (19)	55.8 (48)		

Data was presented in percentages (counts), OR (95% CI) and *p*-value (<0.05). ^(c)^ Chi-square.

**Table 5:** Association of ovarian cancer patients’ HIV serological status with clinical factors.

Clinical Factors	HIV Seropositive 26.7% (23)	HIV Seronegative 73.3% (63)	OR (95% CI)	*p*-Value
Tumor stage % (n)			2.730 (0.566–13.168)	0.335 ^d^
Late stage	24.4 (21)	58.1 (50)		
Early stage	2.3 (2)	15.1 (13)		
Surgical debulking % (n)			1.222 (0.469–3.187)	0.681 ^c^
Yes	12.8 (11)	31.4 (27)		
No	14 (12)	41.9 (36)		
Chemotherapy % (n)			0.858 (0.242–2.242)	0.590 ^c^
No chemo	7 (6)	15.1 (13)		
On chemo	19.8 (17)	58.1 (50)		

Data was presented in percentages (counts), OR (95% CI) and *p*-value (<0.05). ^(c)^ Chi-square and ^(d)^ Fisher’s exact test.

**Table 6:** Association of ovarian cancer patients’ HBV/HIV coinfection status with the clinical factors.

Clinical Factors	HBV/HIV Seropositive 17.4 (15)	HBV/HIV Seronegative 82.6 (71)	OR (95% CI)	*p*-Value
Tumor stage % (n)			1.457 (0.292–7.257)	1.000 ^d^
Late stage	15.1 (13)	67.4 (58)		
Early stage	2.3 (2)	15.1 (13)		
Surgical debulking % (n)			1.562 (0.510–4.779)	0.432 ^c^
Yes	9.3 (8)	34.9 (30)		
No	8.1 (7)	47.7 (41)		
Chemotherapy % (n)			1.279 (0.491–3.329)	0.733 ^d^
No chemo	4.7 (4)	17.4 (15)		
On chemo	12.8 (11)	65.1 (56)		

Data was presented in percentages (counts), OR (95% CI) and *p*-value (<0.05). ^(c)^ Chi-square and ^(d)^ Fishers exact test.

## Data Availability

Not applicable.

## Supplementary Materials

The following supporting information can be downloaded at: https://www.mdpi.com/article/10.3390/idr14030047/s1, Table S1: Analysis of the ovarian cancer patients, Table S2: Analysis of the healthy control subjects.
