# Supplementary material for: The Burden of Hepatitis B, Hepatitis C, and Human Immunodeficiency Viruses in Ovarian Cancer Patients in Nairobi, Kenya
Source: Infect Dis Rep. 2022 Jun 7;14(3):433–45. doi: 10.3390/idr14030047 (PMC9222280; doi:10.3390/idr14030047)
Supplement: Supplementary file 1 [file idr-14-00047-s001.zip › idr-1697750-supplementary.pdf]

Supplementary materials

Table S1. Analysis of the ovarian cancer patients

Study ID	Age	FIGO Stage	Surgery	Chemotherapy	HBV	HCV	HIV	HBV/HIV	HBV/HCV	HIV/HCV	Overall negativity
1	66	late	X	√	neg	neg	neg	neg	neg	neg	neg
2	51	late	X	√	neg	neg	neg	neg	neg	neg	neg
3	28	late	√	√	pos	neg	neg	neg	neg	neg	pos
4	38	late	X	√	neg	neg	neg	neg	neg	neg	neg
5	39	late	X	√	neg	neg	pos	neg	neg	neg	pos
6	45	late	X	√	neg	neg	pos	neg	neg	neg	pos
7	48	late	X	√	neg	neg	neg	neg	neg	neg	neg
8	50	late	X	X	neg	neg	neg	neg	neg	neg	neg
9	49	late	X	√	neg	neg	neg	neg	neg	neg	neg
11	51	late	X	√	pos	neg	pos	pos	neg	neg	pos
12	49	late	X	√	neg	neg	neg	neg	neg	neg	neg
13	69	late	√	√	pos	neg	pos	pos	neg	neg	pos
14	66	late	√	√	neg	neg	neg	neg	neg	neg	neg
15	59	late	√	√	neg	neg	neg	neg	neg	neg	neg
16	45	late	X	√	neg	neg	neg	neg	neg	neg	neg
17	48	late	X	√	neg	neg	neg	neg	neg	neg	neg
18	55	late	√	√	neg	neg	neg	neg	neg	neg	neg
20	60	early	√	√	neg	neg	neg	neg	neg	neg	neg
21	63	late	X	√	neg	neg	neg	neg	neg	neg	neg
22	38	late	X	√	neg	neg	neg	neg	neg	neg	neg
24	43	late	X	√	pos	neg	pos	pos	neg	neg	pos
25	55	late	√	√	neg	neg	neg	neg	neg	neg	neg
26	50	late	X	√	neg	neg	neg	neg	neg	neg	neg
27	48	late	X	√	neg	neg	neg	neg	neg	neg	neg
28	61	late	X	√	neg	neg	neg	neg	neg	neg	neg
29	39	early	X	√	pos	neg	neg	neg	neg	neg	pos

30	20	early	√	√	neg	neg	neg	neg	neg	neg	neg
31	65	late	X	√	neg	neg	neg	neg	neg	neg	neg
32	35	early	√	√	neg	neg	neg	neg	neg	neg	neg
34	81	early	X	√	neg	neg	neg	neg	neg	neg	neg
35	45	late	√	√	pos	neg	neg	neg	neg	neg	pos
36	29	late	X	√	neg	neg	pos	neg	neg	neg	pos
38	45	late	√	√	neg	neg	pos	neg	neg	neg	pos
39	59	early	X	X	pos	neg	pos	pos	neg	neg	pos
40	60	late	√	√	pos	neg	pos	pos	neg	neg	pos
41	27	late	√	√	neg	neg	neg	neg	neg	neg	neg
43	73	early	X	X	pos	neg	pos	pos	neg	neg	pos
44	55	late	√	√	pos	neg	pos	pos	neg	neg	pos
45	34	late	√	√	neg	neg	neg	neg	neg	neg	neg
46	22	late	√	√	neg	neg	neg	neg	neg	neg	neg
47	47	early	√	√	neg	neg	neg	neg	neg	neg	neg
48	48	late	X	X	pos	neg	pos	pos	neg	neg	pos
49	65	early	√	X	neg	neg	neg	neg	neg	neg	neg
50	55	early	√	√	neg	neg	neg	neg	neg	neg	neg
51	29	late	X	√	neg	neg	neg	neg	neg	neg	neg
52	34	late	X	√	pos	neg	pos	pos	neg	neg	pos
53	44	late	X	X	neg	neg	neg	neg	neg	neg	neg
54	44	late	√	√	neg	neg	neg	neg	neg	neg	neg
55	33	late	X	X	neg	neg	neg	neg	neg	neg	neg
56	68	late	X	X	pos	neg	neg	neg	neg	neg	pos
57	50	late	√	√	pos	neg	pos	pos	neg	neg	pos
58	41	early	X	√	neg	neg	neg	neg	neg	neg	neg
59	67	late	X	X	neg	neg	neg	neg	neg	neg	neg
60	44	late	√	√	neg	neg	neg	neg	neg	neg	neg
61	19	early	√	√	pos	neg	neg	neg	neg	neg	pos
63	45	late	√	X	neg	neg	pos	neg	neg	neg	pos
64	70	late	√	√	pos	neg	pos	pos	neg	neg	pos

65	70	late	√	√	pos	neg	pos	pos	neg	neg	pos
66	68	late	√	√	pos	neg	pos	pos	neg	neg	pos
67	33	late	X	X	pos	neg	neg	neg	neg	neg	pos
68	65	late	X	X	neg	neg	neg	neg	neg	neg	neg
69	62	late	X	√	neg	neg	neg	neg	neg	neg	neg
70	28	late	√	√	neg	neg	neg	neg	neg	neg	neg
71	25	early	X	X	neg	neg	neg	neg	neg	neg	neg
72	69	late	X	X	neg	neg	neg	neg	neg	neg	neg
73	72	late	X	X	neg	neg	neg	neg	neg	neg	neg
75	67	late	X	√	pos	neg	neg	neg	neg	neg	pos
76	68	late	X	√	pos	neg	neg	neg	neg	neg	pos
77	65	late	X	√	neg	neg	neg	neg	neg	neg	neg
79	79	early	X	√	neg	neg	neg	neg	neg	neg	neg
80	52	late	X	X	neg	neg	pos	neg	neg	neg	pos
81	38	early	X	X	neg	pos	neg	neg	neg	neg	pos
82	50	late	X	X	pos	neg	pos	pos	neg	neg	pos
84	49	late	√	√	neg	neg	neg	neg	neg	neg	neg
85	52	late	X	√	pos	neg	neg	neg	neg	neg	pos
86	74	late	√	√	pos	neg	neg	neg	neg	neg	pos
87	76	late	√	√	neg	neg	neg	neg	neg	neg	neg
88	28	late	√	√	pos	neg	pos	pos	neg	neg	pos
89	80	late	√	X	neg	neg	neg	neg	neg	neg	neg
10c	24	late	√	√	neg	neg	neg	neg	neg	neg	neg
2c	47	late	√	√	neg	neg	neg	neg	neg	neg	neg
3ca	60	late	√	√	neg	neg	pos	neg	neg	neg	pos
4c	59	late	√	√	neg	neg	neg	neg	neg	neg	neg
5c	21	late	X	√	neg	neg	neg	neg	neg	neg	neg
6c	52	late	√	√	neg	neg	neg	neg	neg	neg	neg
7c	51	late	X	√	neg	neg	pos	neg	neg	neg	pos

Early stage (FIGO stage I and II), late stage (FIGO stage III and IV), √ (done surgery or taken chemotherapy), X (not done surgery or taken chemotherapy), neg (negative) and pos (positive)

Table S2. Analysis of the healthy control subjects

Study ID	Age	HBV	HCV	HIV	HBV/HIV	HBV/HCV	HIV/HCV	Overall negativity
2	38	neg	neg	neg	neg	neg	neg	neg
3	74	neg	neg	neg	neg	neg	neg	neg
4	26	neg	neg	neg	neg	neg	neg	neg
5	39	neg	neg	neg	neg	neg	neg	neg
6	32	neg	neg	neg	neg	neg	neg	neg
7	28	neg	neg	neg	neg	neg	neg	neg
8	24	neg	neg	neg	neg	neg	neg	neg
9	18	neg	neg	neg	neg	neg	neg	neg
11	21	neg	neg	neg	neg	neg	neg	neg
12	28	neg	neg	neg	neg	neg	neg	neg
13	25	neg	neg	neg	neg	neg	neg	neg
14	49	neg	neg	neg	neg	neg	neg	neg
15	31	neg	neg	neg	neg	neg	neg	neg
16	53	neg	neg	neg	neg	neg	neg	neg
17	33	neg	neg	neg	neg	neg	neg	neg
18	59	pos	neg	neg	neg	neg	neg	pos
19	26	neg	neg	neg	neg	neg	neg	neg
20	56	neg	neg	neg	neg	neg	neg	neg
21	33	neg	neg	neg	neg	neg	neg	neg
22	34	neg	neg	neg	neg	neg	neg	neg
24	36	neg	neg	neg	neg	neg	neg	neg
25	34	neg	neg	neg	neg	neg	neg	neg
26	25	neg	neg	neg	neg	neg	neg	neg
27	40	neg	neg	neg	neg	neg	neg	neg
28	23	neg	neg	neg	neg	neg	neg	neg
29	47	neg	neg	pos	neg	neg	neg	pos
30	55	neg	neg	neg	neg	neg	neg	neg

31	30	neg	neg	neg	neg	neg	neg	neg
32	34	neg	neg	neg	neg	neg	neg	neg
33	34	neg	neg	neg	neg	neg	neg	neg
34	46	neg	neg	neg	neg	neg	neg	neg
35	50	pos	neg	neg	neg	neg	neg	pos
36	34	neg	neg	neg	neg	neg	neg	neg
39	50	neg	neg	neg	neg	neg	neg	neg
40	36	neg	neg	neg	neg	neg	neg	neg
41	56	neg	neg	neg	neg	neg	neg	neg
42	42	neg	neg	neg	neg	neg	neg	neg
43	37	neg	neg	neg	neg	neg	neg	neg
44	34	neg	neg	neg	neg	neg	neg	neg
45	47	neg	neg	neg	neg	neg	neg	neg
46	43	neg	neg	neg	neg	neg	neg	neg
11b	27	neg	neg	neg	neg	neg	neg	neg
1b	30	neg	neg	neg	neg	neg	neg	neg
1H	25	neg	neg	neg	neg	neg	neg	neg
2H	49	neg	neg	neg	neg	neg	neg	neg
3H	35	neg	neg	neg	neg	neg	neg	neg
4H	46	neg	neg	neg	neg	neg	neg	neg
5H	47	neg	neg	neg	neg	neg	neg	neg
6H	33	neg	neg	neg	neg	neg	neg	neg
7H	42	neg	neg	pos	neg	neg	neg	pos

neg (negative) and pos (positive)
